# The Rational Treatment of Serous Iritis

**Published:** 1891-07-18

**Authors:** 


### OPHTHALMOLOGY.

### THE RATIONAL TREATMENT OF SEROUS IRITIS.

Up to the present time the treatment of serous iritis has been unsatisfactory, partly, no doubt, on account of the inherent tendency of the disease to be chronic, but also because the nature of the disease has not been properly understood, and so the treatment has not been based on rational principles. Recent investigations have thrown some light on the physiology and pathology of the ciliary region, and we are now able to understand better the nature of the morbid processes going on in *aquo-capsulitis*, or, as it is generally called, *serous iritis*.

At a recent meeting of the Ophthalmological Society, Mr. Treacher Collins described a method of bleaching sections of the ciliary region with chlorine so as to remove the pigment and render the structure and arrangement of the cells more visible. By this means he was able to demonstrate, what could not be so easily seen before, that the cells covering the ciliary region have the characters of secreting cells, and are arranged so as to form follicles or crypts. These, he shows are really glands which secrete the aqueous and provide nourishment for the vitreous. In eyes lost by serous iritis he finds these glands increased in size and the epithelium irregular and proliferated. (It is interesting to note that these cells, which are morphologically undifferentiated cells of the optic vesicle, both in structure and function bear the same relation to the retina that the cells covering the choroidal plexus do to the brain.)

Nicati of Marseilles (*Archives d'Ophtal., Nov.*, 1890—*April*, 1891) has made most important researches in this matter. He lays stress on the fact that this gland (the cells lying between the ora serrata and the root of the iris) is largely situated between two impermeable structures, the hyaline membrane of the vitreous internally and the limiting membrane of the choroid externally, so that any secretion from it can only go through the pupil and into the anterior chamber. Arrived there it cannot get through Descemet's membrane, and its only escape is by filtering into the lymphatics of the anterior surface of the iris. Nicati gives three proofs that the aqueous is secreted by the cells covering the ciliary region. (1) He successfully repeated Deutschmann's experiment, the whole of the ciliary body was removed under antiseptic precautions which caused complete cessation of the secretion of the aqueous. (2) He found a cat with persistent pupillary membrane, in which the anterior chamber was rather shallow, whilst the posterior chamber was of enormous depth, the lens being pushed back by the fluid. This shows that the aqueous comes from the ciliary region. That it is *secreted* he proved by (3) injecting fluorescine under the skin of a rabbit; this caused no staining of the aqueous or of the cells covering the ciliary region, but the blood and tissues beneath the epithelium were deeply stained. He further found that the secretion was regulated by the action of the sympathetic; irritation of the cornea or iris causing increased secretion and increased tension, whilst removal of the iris, or part of it, diminished the tension. From these facts it is clear that whenever the secretion of the aqueous is increased or the filtration apparatus of the iris impaired an accumulation of aqueous will result with increased tension. This will react upon the ciliary region and cause œdema of the membrane on which the ciliary gland lies, and of the extension of that membrane the chorio-capillaris. As a matter of fact, we have just these causes at work in serous iritis. Not only is the iris engorged with blood and rendered almost impermeable by inflammatory exudation (or in cases of long duration by partial atrophy), but also, as Collins has shown, there is a *catarrhal* condition of the ciliary gland.

In the light of these facts the lines of treatment indicated are as follows:—First, to diminish the engorgement of the iris by affording it *complete* rest, shielding both eyes from light and using atropine. The atropine should be used dilute and not more often than is necessary to keep the iris paralysed. (Eserine would not reduce the tension because it increases the vascularity of the iris.) Secondly, to improve the general health of the patient by tonics (iron, quinine, arsenic), and, what in some cases is most important, change of air. Thirdly, to relieve the tension of the eye, and so the œdema of the ciliary body and chorio-capillaris. This will often result from the use of the treatment recommended above, but if the tension persists recourse must be had to either paracentesis of the cornea, the wound being reopened daily if necessary, or to iridectomy. The former should always be tried first and persisted in for some time (weeks, or perhaps months), for in performing iridectomy there is always the danger that the irritation produced may raise the tension more than it is reduced by the removal of part of the iris.

